# Incident Rheumatoid Arthritis Following Statin Use: From the View of a National Cohort Study in Korea

**DOI:** 10.3390/jpm12040559

**Published:** 2022-04-01

**Authors:** Mi Jung Kwon, Joo-Hee Kim, Ji Hee Kim, Hye-Rim Park, Nan Young Kim, Sangkyoon Hong, Hyo Geun Choi

**Affiliations:** 1 Department of Pathology, Hallym University Sacred Heart Hospital, Hallym University College of Medicine, Anyang 14068, Korea; mulank@hanmail.net (M.J.K.); hyerim@hallym.or.kr (H.-R.P.); 2 Division of Pulmonary, Allergy, and Critical Care Medicine, Department of Medicine, Hallym University Sacred Heart Hospital, Hallym University College of Medicine, Anyang 14068, Korea; luxjhee@gmail.com; 3 Department of Neurosurgery, Hallym University Sacred Heart Hospital, Hallym University College of Medicine, Anyang 14068, Korea; kimjihee.ns@gmail.com; 4 Hallym Institute of Translational Genomics and Bioinformatics, Hallym University Medical Center, Anyang 14068, Korea; honeyny78@gmail.com (N.Y.K.); kyoons@gmail.com (S.H.); 5 Department of Otorhinolaryngology-Head & Neck Surgery, Hallym University Sacred Heart Hospital, Hallym University College of Medicine, Anyang 14068, Korea

**Keywords:** rheumatoid arthritis, statin, nested case–control study, health insurance claim data, lipophilic statin, hydrophilic statin

## Abstract

Safety issues regarding the potential risk of statins and incident rheumatoid arthritis (RA) have been raised, but the existing data are largely based on Caucasian populations, and continue to have biases and require further validation in Asian populations. Here, we aimed to verify the risk of RA depending on the duration of previous statin use and statin types using a large-scale, nationwide database. This study enrolled 3149 patients with RA and 12,596 matched non-RA participants from the national health insurance database (2002–2015), and investigated their statin prescription histories for two years before the index date. Propensity score overlap-weighted logistic regression was applied after adjusting for multiple covariates. The prior use of any statins and, specifically, the long-term use of lipophilic statins (>365 days) were related to a lower likelihood of developing RA ((odds ratio (OR) = 0.73; 95% confidence intervals (CI) = 0.63–0.85, *p* < 0.001) and (OR = 0.71; 95% CI = 0.61–0.84, *p* < 0.001), respectively). Subgroup analyses supported these preventive effects on RA in those with dyslipidemia, independent of sex, age, smoking, alcohol use, hypertension, and hyperglycemia. Hydrophilic statin use or short-term use showed no such associations. Our study suggests that prior statin use, especially long-term lipophilic statin use, appears to confer preventive benefits against RA.

## 1. Introduction

Rheumatoid arthritis (RA) is an intractable chronic systemic inflammatory and autoimmune disease, with rapid onset of clinically significant functional disability and related early mortality [1]. RA is present worldwide, accounting for approximately 1% of the global population and 0.27–1.85% of the Korean population [2,3]. Although RA can develop at any age, the incidence likelihood increases with age, especially after 50 years [4]. Considering increasing aging population with prolonged life expectancy worldwide, the prevalence of RA is expected to increase [5,6]. In Korea, RA is becoming a public health challenge in relation to higher health care expenditure, increased financial vulnerability, and higher risks for developing serious comorbidities for individuals with RA than the general population [7]. One-third of patients suffering from RA quit working within two years, and half are incapable of working within 10 years [5,7,8]. The most effective way to reduce the incidence of RA may be primary prevention [9]. The specific cause of RA cannot be pinpointed, but genetic and environmental factors that are important for developing the disease include advanced age, female sex, a family history of RA, genetic factors, smoking, obesity, diet, and stress [10,11]. In fact, a recent randomized double-blinded clinical trial ascertained lifestyle modifications, including a prebiotic-rich diet, as having substantially beneficial effect on the inflammatory regulation of RA [11]. Since there is no established way of preventing RA absolutely, it is worthwhile to uncover and lessen any possible modifiable risk factors as an effective step towards RA prevention, in order to mitigate the emerging socioeconomic burden of this disorder.

Statins are a mainstay of primary prevention for atherosclerotic cardiovascular disease, playing a role as competitive inhibitors against 3-hydroxy-3 methyl-glutaryl-coenzyme A reductase, a crucial enzyme that regulates cholesterol synthesis and lipid metabolism [12]. The expansion of the clinical use of statins has been attempted given their cholesterol-independent pleiotropic features, including anti-inflammatory, immunomodulatory, antioxidant, antimicrobial, antifungal, and anticancer properties [12,13,14]. In particular, the anti-inflammatory property of statins has been anticipated to have a preventive effect in persons at high risk for RA, especially for first-degree or second-degree relatives of RA patients, because persistent inflammation is considered one of the major pathogenic mechanisms of the occurrence of RA. Meanwhile, the immunomodulatory efficacy of statins has drawn attention to safety issues of the rare but serious risk of triggering the development of autoimmune disorders, including a spectrum of rheumatic diseases [15]. It has been proposed that statins may directly modulate T cell activation and induce an altered immune response via statin binding sites on T cells, according to in vitro and animal models of arthritis [16], which may disrupt the immune systemic balance and cause predisposing conditions that can make people vulnerable to autoimmune diseases, including RA [17].

Despite the tremendous increase in the concern for the hazard-to-benefit ratio of statins over the last decade, there remains much controversy regarding the relationship between statin use and the risk of developing RA, with no more than seven primary studies specific to this topic [8,17,18,19,20,21,22]. However, these publications are mainly based on Western populations [8,17,18,19,20,21,22], which may be unable to be generalized, and they appear to have certain limitations, being devoid of universal nationwide healthcare databases covering nearly the whole population. A recent meta-analysis reported no significant difference in the incidence rate of RA between statin users and nonusers, based on only four eligible studies with a high degree of heterogeneity [23], indicating that further validation is needed. Since statins are widely used worldwide, it is inevitably important to clarify their effects on the risk of RA development by means of further validation in the Asian population.

Herein, we supposed that statin use might affect the subsequent occurrence of RA depending on patient factors, including sex, age, social or economic status and comorbid conditions, and the factors related to statin use duration or statin types. Using a nationwide health insurance database, we investigated the likelihood of RA in patients who had previously been administered statins compared with the matched control group by adjusting for potential confounding factors.

## 2. Patients and Methods

### 2.1. Study Design and Participants

The Ethics Committee (IRB No. 2019-10-023) approved this study, and written informed consent was waived. This retrospective cohort study, based on a nested case–control design, used the Korean National Health Insurance Service-Health Screening Cohort (KNHIS-HSC) database, which provides population-based data on a representative sample cohort of the Korean population for research purposes, as previously described [24].

All data on patients diagnosed with RA were extracted from medical claims using International Classification of Diseases, tenth revision (ICD-10) codes and prescription codes. A total of 4228 RA participants at baseline were included from 514,866 adults aged 40 years and above, with 615,488,428 medical claim codes from 2002 through 2015. Signs and symptoms of RA may begin stealthily and amass over months (>6 months) [25], so it may be plausible that certain cases were in a state of subclinical illness for some time before their diagnosis. Such clinically unobvious cases of RA may be included in this study. To select RA participants who were diagnosed first, we excluded those diagnosed within one year from beginning the study, which is referred to as the “1-year washout period”, to eliminate insidious cases (1-year washout period, *n* = 1079). The final RA group included only patients diagnosed from 1 January 2003.

Data for the comparison of individuals without a history of RA were also extracted from the database (*n* = 510,638) using a random number order to reduce possible selection bias. We excluded patients who had ever been assigned any RA diagnosis claim code or who had any RA-related medication history (*n* = 78,040).

**Figure 1:**
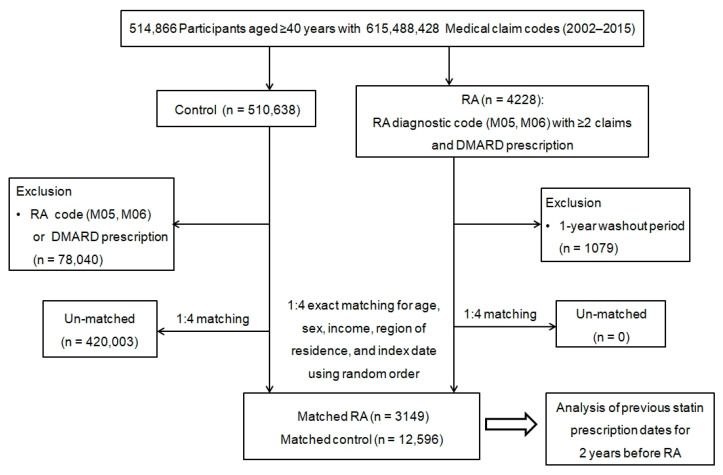
A schematic illustration of the participant selection process.

To minimize the differences between both groups’ baseline characteristics, propensity score matching was performed based on age, sex, income, and area of residence. Hence, patients with RA were individually matched with control participants based on similar propensity score values. The index date of every patient with RA was defined as the day on which the ICD-10 codes for RA were electronically assigned in the health insurance datasets. The index date of a participant in the comparison group was determined as the index date of their matched patient with RA. During the matching process, 420,003 unmatched control participants were excluded. Finally, 3149 patients with RA were matched with 12,596 control participants at a 1:4 ratio (Figure 1). Then, we analyzed the previous histories of statin use over the two years before the index dates in both cohort groups.

### 2.2. Exposure (Statin)

Participants who were using statins for the first time were included, and previous histories of statin were identified based on prescription data. The summation of the total prescription dates of statins was considered the continuous variable, and was assessed for 2 years before the index dates because the effects of statins can last for 2 years [26].

We investigated the statin duration effect for RA using conditional logistic regression in the following categories: <90 days, 90–365 days, and >365 days. Statin users were considered patients who had had statin prescriptions for a minimum of 90 days [26,27]. The patients who were deemed statin nonusers had prescriptions for <90 days, those deemed short-term users had prescriptions from 90–365 days, and those deemed long-term users had prescriptions for >365 days, as previously described [13,26,27].

The statins surveyed in this study were categorized as lipophilic statins (atorvastatin, simvastatin, lovastatin, and fluvastatin) and hydrophilic statins (pravastatin and rosuvastatin) in order to investigate potential impacts according to the effects of lipids of the statin types on developing RA.

### 2.3. Outcome (Rheumatoid Arthritis)

RA was defined according to the presence of at least one corresponding RA diagnostic code out of the ICD-10 codes (M05 (seropositive rheumatoid arthritis), M06 (other rheumatoid arthritis)), assigned following more than 2 clinic visits, and a prescription for the related biological agent or any disease-modifying anti-rheumatic drugs (DMARDs) under the RA diagnostic code, for which a diagnostic approach in a claim database had been proven to reach high sensitivity and accuracy rates and to have a positive predictive value of 96.46%, 90.33% and 92.35%, respectively [28]. The primary outcomes were the incidence of RA during the designated periods depending on the statin type.

### 2.4. Covariates

The participants were divided into 10 age groups based on 5-year intervals from the age of 40, and 5 income groups (class 1 (lowest income) to class 5 (highest income)). The region of residence was grouped into urban and rural areas following our previous study [29]. Tobacco smoking, alcohol consumption, and obesity using body mass index (kg/m^2^) were categorized in the same way as in our previous study [24]. The records of total cholesterol (mg/dL), systolic blood pressure (mmHg), diastolic blood pressure (mmHg), fasting blood glucose (mg/dL), and hemoglobin (g/dL) levels were used. The Charlson Comorbidity Index (CCI) has been used widely to measure disease burden using 17 comorbidities as continuous variables (0 (no comorbidities) through 29 (multiple comorbidities)). In our study, we excluded rheumatoid disease from the CCI score. Regarding statins, dyslipidemia (E78) was assigned if participants were treated ≥ 2 times. We adjusted the potential confounding factors of age, sex, income, residence, obesity, smoking, alcohol, systolic or diastolic blood pressure, fasting blood glucose, total cholesterol, hemoglobin, dyslipidemia, statin types, and CCI scores using overlap weighted models by multivariable conditional logistic regression (when we analyzed lipophilic statin, hydrophilic statin was adjusted as the covariate, and vice versa).

### 2.5. Statistical Analyses

The propensity score was calculated by multivariable logistic regression with the aforementioned baseline covariates. A greedy nearest-neighbor matching algorithm was applied during propensity score matching to form pairs of patients with RA and control participants whose propensity scores were closest to those of the patients [30]. The standardized difference was used to compare the rate of general characteristics between the groups. To assess bias reduction, balance between the groups was checked based on absolute standardized differences in the covariates before and after matching. An absolute standardized difference of <0.20 indicated good balance for a particular covariate [30]. The categorical data are summarized as numbers and percentages. Continuous data are depicted as the mean and standard deviation.

We conducted propensity score overlap weighting to achieve an exact balance and optimize the precision [31]. Overlap weighting was calculated to between 0 and 1, whereby RA participants’ data were weighted by the probability of a 1-propensity score and control participants’ data were weighted by the probability of a 0-propensity score. Propensity score overlap-weighted multivariable logistic regression analysis was used in crude (unadjusted) and overlap-weighted models (adjusted for age, sex, income, region of residence, SBP, DBP, fasting blood glucose level, total cholesterol level, hemoglobin level, obesity, smoking, alcohol consumption, dyslipidemia history, and CCI scores) to calculate the crude and adjusted odds ratios (ORs) together with their 95% confidence intervals (CIs). Subgroup analyses were conducted to assess all covariate variables. Two-tailed analyses were performed, and significance was defined as *p*-values less than 0.05. SAS version 9.4 (SAS Institute Inc., Cary, NC, USA) was used for statistical analyses.

## 3. Results

### 3.1. Baseline Characteristics of the Study Participants

After propensity score matching, 3149 participants with RA and 12,596 non-RA control groups were included. Among the 33,149 RA participants, men accounted for 26.83% and women accounted for 73.17%, with a predominance of nonsmokers (82.09%) and fewer alcohol drinkers (80.57%). Before applying the overlap weighting adjustment, the baseline characteristics between the two cohorts were slightly imbalanced with regard to obesity status, dyslipidemia, hemoglobin level, fasting blood glucose level, blood pressure, smoking status, alcohol consumption, CCI score, or total cholesterol level. After applying the overlap weighting adjustment, the standardized mean differences were reduced to the minimum, and the balance between the groups became the same (standardized difference = 0.00) (Table 1).

The proportions of patients in the designated periods of statin use depending on statin type (any statin vs. lipophilic statin vs. hydrophilic statin) were also similar between the RA and control groups (standardized difference ≤ 0.2).

### 3.2. Odds Ratios of the Incidence of RA for the Duration of Use and Types of Statins

We estimated the odds for subsequent RA depending on either the designated period of previous use of any statin, or statin type (Table 2). After full adjustment in the overlap-weighted model, the use of either any statin or lipophilic statin was related to decreased odds for developing RA when using a long-term period (>365 days) ((OR = 0.73; 95% CI = 0.63–0.85, *p* < 0.001) and (OR = 0.71; 95% CI = 0.61–0.84, *p* < 0.001), respectively). Hydrophilic statin use had no statistically significant association with subsequent RA.

**Figure 2:**
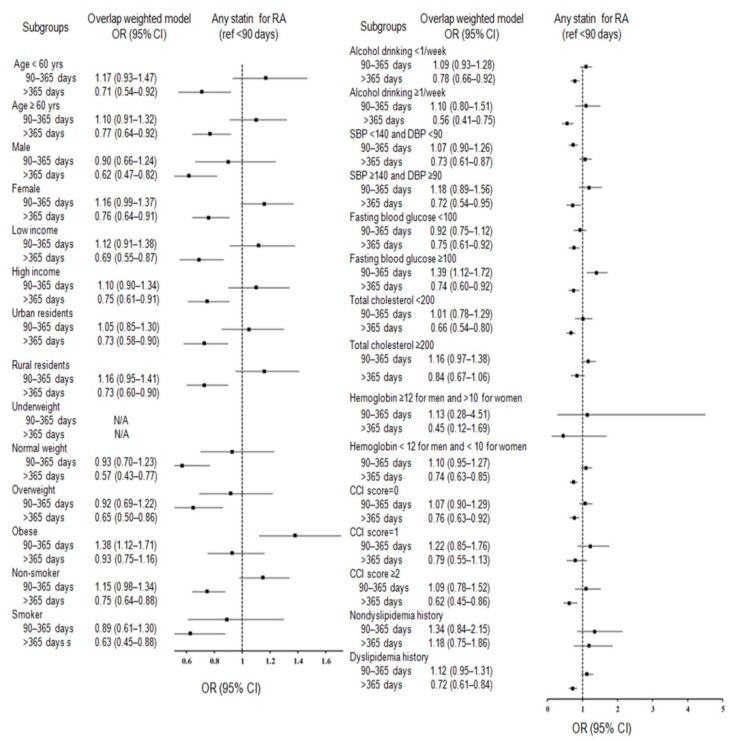
Forest plots depicting the association between use duration of any statin and a subsequent risk of incident rheumatoid arthritis (RA) in each subgroup.

**Figure 3:**
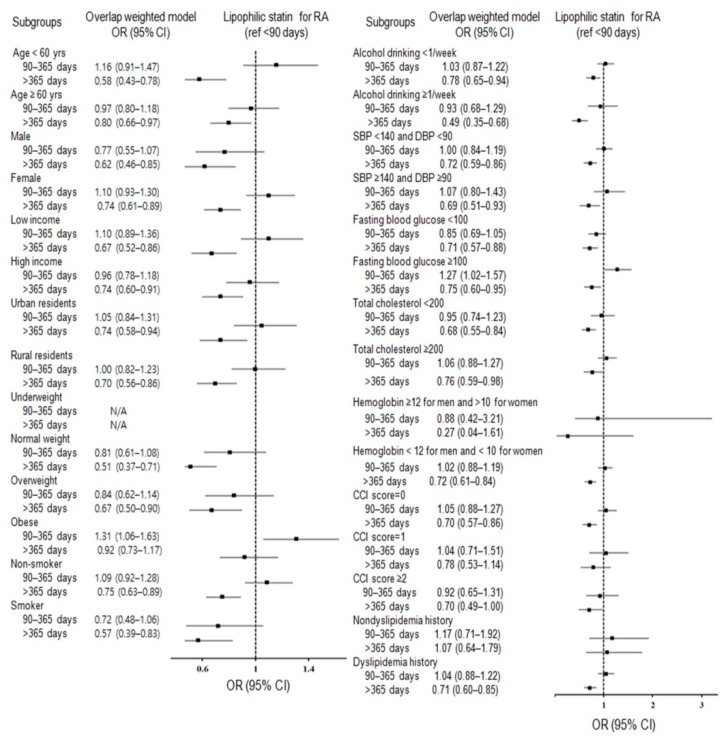
Forest plots depicting the association between use duration of lipophilic statin and a subsequent risk of incident rheumatoid arthritis (RA) in each subgroup.

Comprehensive subgroup analyses in terms of 29 baseline covariates were performed. Consequently, subgroup analyses for any statin (Figure 2; Supplementary Table S1) and lipophilic statin (Figure 3; Supplementary Table S2) supported the significance of statin use for >365 days with decreased odds for RA in both men and women, regardless of hyperglycemia, income, age, residence, sex, smoking, hypertension, and alcohol consumption, and in patients with normal weight and overweight, patients with anemia, patients with CCI scores 0 or ≥2, and patients with dyslipidemia history.

However, hydrophilic statin use had no statistically significant association with subsequent RA (Supplementary Figure S1; Supplementary Table S3).

## 4. Discussion

The present nationwide cohort study demonstrates that prior statin use, particularly lipophilic statin use over a long-term period (>365 days), is associated with reduced odds of RA in both men and women, and is independent of age, smoking, alcohol consumption, hypertension, and hyperglycemia. The findings have important implications for the prevention potential of statin use, even in elderly individuals over 65 years of age and those with hypertension or hyperglycemia. A more personalized approach toward preventive healthcare is a major paradigm shift in current medical practices, and would encourage individuals at high risk for RA to be more active in their health management, which is a prerequisite for improving public health [6,32].

There is growing interest in the prediction and prevention of RA to facilitate early intervention, and stratified approaches for people at risk of developing RA [9,33]. However, the difficulties involved in recruiting eligible participants in RA-prevention trials seem to be higher than in trials with other disease, largely based on the anxiety and uncertainty of the effect of statins [9,34]. Our results may be clinically useful in helping individuals at risk for RA to somewhat reduce their negative perceptions of statins and their feelings of anxiety, which could cause uncertain harm. This nationwide cohort study suggests that long-term preventive statin use prior to RA onset could help prevent or slow the development of the disease. In our analysis, patients with any previous long-term statin and lipophilic statin use exhibited 27% and 37% lower reductions in the odds for RA, respectively. These results regarding the disease prevention effect of statins are in line with five previous studies [8,18,20,22,27], of which three studies were conducted in the UK [8,18,22]. Although the studies performed in the UK used slightly different primary care medical records, they showed a similar protective effect of statins related to a 41% lower risk for RA in hyperlipidemic patients (95% CI = 0.37–0.96) [18], a 24% lower risk in diabetic patients (95% CI = 0.66–0.88) for both men and women [8], and a 23% lower risk for patients using high-dose statins (95% CI = 0.63–0.95) [22]. Another large study in Israel with 211,627 new statin users accentuated the protective effect against RA in persistent statin users, with use for at least one year [20], wherein the effect was greater for individuals at a younger age, highlighting the importance of early prevention by long-term statin use, which was similar to our study. The decreased odds of contracting RA in these patients are comparable with the present findings that the beneficial impact of long-term statin use was sustained even after adjusting for a history of dyslipidemia, fasting glucose level, and total cholesterol level.

The majority of the overall existing data indicate that statin use does not significantly increase RA risk [8,13,18,20,21,22,23,27,35]. Only two studies based on different national databases conducted by the same author group reported that statin use increased the OR of RA by 1.71-fold (95% CI = 1.16–2.53) and 1.39-fold (99% CI = 1.01–1.90) in the Netherlands and UK datasets, respectively [17,19]. The authors accessed the UK Clinical Practice Research Datalink [17], which is the same data source, showing the association of statin use with reduced odds for RA, as was mentioned above [22]; ironically, it disclosed the opposite conclusion regarding the hazardous effect of statins on incident RA [17], contrasting the former UK study [22]. The authors declared an increase in odds for RA for very recent statin users, but not for all statin users [17]. The discrepancies might be attributable to the different aims of the studies, with considerable variations in the enrolled populations, age range inclusion criteria (≥40 years [13,17,18,19,22], ≥30 years [27,35], or ≥18 years [8,20,21]), sorting methods for RA patients, follow-up duration, categories of periods of use and statin types, as well as possible uncalculated confounders [8,13,23,35]. To avoid any possible selection bias and heterogeneity in the current research, we followed strict criteria for sorting RA patients according to a previous evidence-based study [28], the diagnostic approach of which was proven to reach reliable validity for identifying RA patients in a claim database [28]. To minimize potential confounding effects, we applied a methodologically preferable study design using nationwide well-organized data, and comprehensively adjusted for possible confounders. Following this, we were able to determine the preventive potential of prior statin use on RA.

The underlying mechanism by which statins may reduce the occurrence of RA is unclear. Inflammation and an imbalance in inflammatory cytokines are perceived to play a central role in the underlying pathogenesis of RA [16,36]. Because RA also accelerates vascular risk [37], RA and atherosclerosis seem to share a common pathogenic basis in terms of inflammation, immunological processes, and abnormal lipid profiles [37,38,39]. Activated inflammatory and immunological activity, including alterations in macrophages and T and B cell functions, together with their proinflammatory cytokines, drives RA and plays a main role in the aggravation of atherosclerosis [38]. The strong anti-inflammatory features of statins may relieve pathogenic inflammatory responses in RA. Experimental evidence suggests that statins repress the production of proinflammatory cytokines in RA-derived synovial fibroblasts and animal models [14,40]. Statins also inhibit the induction of major histocompatibility complex-II expression, which is central to controlling immune response and RA susceptibility [14,32], which may stabilize the immunomodulatory activation and prevent it from progressing to RA [14]. Indeed, some of the risk factors involved in RA, including smoking, obesity, aging, and stress, can affect the immune system by accelerating oxidative stress in the body, provoking inflammation, and enhancing apoptosis [10]. The pleiotropic effects of statin use prior to the development of RA may stabilize predisposing risk factors, which might contribute to preventing the pathogenic process from progressing to RA. Surprisingly, the administration of statins itself may influence people to considerably modify their health behaviors, including undertaking physical activity, nutritional intervention, or medication, which might improve baseline health [41].

We found that prior use of lipophilic statins was related to a decreased likelihood of RA, but no such association was found with hydrophilic statin use. Scarce information is available on the preventive effects of statin types against RA. Possible explanations may be found in a few experimental and clinical studies. Atorvastatin (a lipophilic statin) has shown clinically apparent anti-inflammatory effects through a decline in plasma inflammatory markers, improved articular swelling [39], and a significant decrease in intraplaque rupture by promoting vascular maturation [42]. Simvastatin (a lipophilic statin) suppresses IFN-gamma release from mononuclear cells and proinflammatory cytokines produced by activated macrophages [40]. In contrast, pravastatin (a hydrophilic statin) reportedly did not affect the improvement of RA [18]. Lipophilic statins seem to have more beneficial effects anti-inflammatory activity, immune suppression, and vascular remodeling, which may be more effectively associated with a reduction in the likelihood of RA than hydrophilic statins. Nonetheless, a recent clinical trial unfortunately failed to show the preventive effects of atorvastatin in high-risk individuals with RA, mainly hindered by the low recruitment for the high-risk RA group [34], of which the result seems to be inconclusive and still needs to be verified [34].

It is noticeable that the proportion of RA tended to be higher in participants with the highest income in the present results regarding Korean nationwide data. According to a global burden of RA study in 2017, East Asia, high-income North America, and North Africa and the Middle East show the most increasing trends in age-standardized incidence rates. In national-level analysis, a non-linear association is observed between socio-demographic index and the burden of RA, regardless of whether one is in the most developed or least developed countries [6]. However, these socio-economic imbalances may result in diagnosis in different phases of disease progress, and different therapeutic outcomes; for example, in terms of healthcare regimen affiliation or accessibility to medical institutes [43]. The high-income population, who can afford wider-covering healthcare services, has been reported to be associated with earlier detection, lower disease severity, and better outcomes when compared to those in other lower income groups [43].

The main strengths of the present study include the exactly balanced cohorts based on well-organized nationwide healthcare databases, which can minimize selection bias and mimic randomized trials. This study was first validated based on Asian cohorts, and lends support to the protective effect of statin use on incident RA. Because the KNHIS-HSC database includes every hospital and clinic in the whole nation without exception, no medical history was lost in the follow-up, which implies the generalizability of our data. Using universally applied nationwide data may overcome the limited nature of data from previous studies based on relatively region-based or selected medical records. We comprehensively considered possible confounders, including 29 covariates, which is far more than previous studies. To minimize selection bias and confounding effects, the comparison group was randomly chosen by propensity score matching, and the overlap weighting method was used to adjust the variables. A large-scale, sophisticated observational study that reduces confounding factors may be a reasonable alternative to assess statins’ impacts on incident RA.

Several limitations of the present study should be taken into account. First, no specifics were gathered about the family history of RA—one of the most important risk factors for developing RA. Second, there are also no figures on the presence or absence of autoantibodies (immunoglobulin M rheumatoid factor/anticitrullinated protein antibodies), which are clinically important in the identification and treatment follow-up of RA. Third, accordingly, the identification of RA was based on ICD-10 codes, which may not comprise the complete range of RA; however, the majority of previous studies identified RA using their own or official diagnostic codes, and we followed the previously validated sorting method [28]. Fourth, we used prescription days, but actual medication intake could not be monitored in this study. The patient compliance with medication intake cannot be measured. Third, although we tried to include confounding factors as adjustments, possible confounding effects may not have been perfectly eliminated, which is a limitation of this retrospective study. The nonidentical sizes of the hydrophilic and lipophilic statin user groups are examples. Since many participants used uncontrolled hydrophilic or lipophilic statins, we adjusted each medication in the analysis as covariates to try to remove possible confounders. Fifth, information on the genetic results, comedications, diet, and type of alcohol was lacking in the health insurance data, and was not taken into account.

## 5. Conclusions

Overall, this nationwide cohort study provides updated epidemiological evidence supporting the positive prevention effects of prior statin use over a long-term period on the reduced likelihood of incident RA, especially for lipophilic statins. Our findings offer an important reference evidence base, providing information on the preventive potential of statin use over a long-term period against RA, and contribute to relieving the reluctance to use statins, especially in individuals at risk for RA.

## Figures and Tables

**Table 1:** General characteristics of participants.

Characteristics	before Overlap Weighting Adjustment			after Overlap Weighting Adjustment		
	RA (*n* = 3149)	Control (*n* = 12,596)	SMD	RA (*n* = 2499)	Control (*n* = 2499)	SMD
Age (%)			0.00			0.00
40–44	109 (3.46%)	436 (3.46%)		86 (3.44%)	86 (3.44%)	
45–49	365 (11.59%)	1460 (11.59%)		289 (11.58%)	289 (11.58%)	
50–54	682 (21.66%)	2728 (21.66%)		541 (21.67%)	541 (21.67%)	
55–59	591 (18.77%)	2364 (18.77%)		470 (18.79%)	470 (18.79%)	
60–64	552 (17.53%)	2208 (17.53%)		439 (17.56%)	439 (17.56%)	
65–69	428 (13.59%)	1712 (13.59%)		340 (13.60%)	340 (13.60%)	
70–74	248 (7.88%)	992 (7.88%)		196 (7.86%)	196 (7.86%)	
75–79	134 (4.26%)	536 (4.26%)		106 (4.24%)	106 (4.24%)	
80–84	35 (1.11%)	140 (1.11%)		28 (1.1%)	28 (1.1%)	
85+	5 (0.16%)	20 (0.16%)		4 (0.16%)	4 (0.16%)	
Sex (%)			0.00			0.00
Male	845 (26.83%)	3380 (26.83%)		668 (26.72%)	668 (26.72%)	
Female	2304 (73.17%)	9216 (73.17%)		1831 (73.28%)	1831 (73.28%)	
Income (%)			0.00			0.00
1 (lowest)	529 (16.8%)	2116 (16.8%)		419 (16.76%)	419 (16.76%)	
2	476 (15.12%)	1904 (15.12%)		379 (15.15%)	379 (15.15%)	
3	541 (17.18%)	2164 (17.18%)		430 (17.2%)	430 (17.2%)	
4	669 (21.24%)	2676 (21.24%)		531 (21.23%)	531 (21.23%)	
5 (highest)	934 (29.66%)	3736 (29.66%)		741 (29.66%)	741 (29.66%)	
Region of residence (%)			0.00			0.00
Urban	1360 (43.19%)	5440 (43.19%)		1080 (43.21%)	1080 (43.21%)	
Rural	1789 (56.81%)	7156 (56.81%)		1419 (56.79%)	1419 (56.79%)	
Obesity † (%)			0.05			0.00
Underweight	61 (1.94%)	283 (2.25%)		50 (1.98%)	50 (1.98%)	
Normal	1201 (38.14%)	4567 (36.26%)		943 (37.75%)	943 (37.75%)	
Overweight	826 (26.23%)	3360 (26.68%)		658 (26.31%)	658 (26.31%)	
Obese I	970 (30.8%)	3974 (31.55%)		775 (31.01%)	775 (31.01%)	
Obese II	91 (2.89%)	412 (3.27%)		74 (2.95%)	74 (2.95%)	
Smoking status (%)			0.04			0.00
Nonsmoker	2585 (82.09%)	10,494 (83.31%)		2061 (82.46%)	2061 (82.46%)	
Past smoker	217 (6.89%)	770 (6.11%)		167 (6.67%)	167 (6.67%)	
Current smoker	347 (11.02%)	1332 (10.57%)		272 (10.87%)	272 (10.87%)	
Alcohol consumption (%)			0.05			0.00
<1 time a week	2537 (80.57%)	9883 (78.46%)		2004 (80.21%)	2004 (80.21%)	
≥1 time a week	612 (19.43%)	2713 (21.54%)		495 (19.79%)	495 (19.79%)	
SBP (Mean, SD)	125.13 (16·37)	126.10 (17.48)	0.06	125.30 (14.60)	125.30 (7.67)	0.00
DBP (Mean, SD)	77.52 (10.79)	78.08 (11.05)	0.05	77.62 (9.61)	77.62 (4.87)	0.00
FBG (Mean, SD)	96.85 (27.20)	99.37 (29.65)	0.03	97.30 (25.12)	97.30 (11.03)	0.00
Total cholesterol (Mean, SD)	200.62 (38.09)	201.91 (38.70)	0.09	200.87 (33.93)	200.87 (17.12)	0.00
Hemoglobin (Mean, SD)	13.22 (1.42)	13.37 (1.41)	0.11	13.25 (1.26)	13.25 (0.64)	0.00
CCI score (Mean, SD)	0.92 (1.52)	0·66 (1.40)	0.04	0.71 (1.33)	0.71 (0.65)	0.00
Dyslipidemia history (%)	1553 (49.32%)	5598 (44.44%)	0.1	1207 (48.32%)	1207 (48.32%)	0.00
Any statin (%)			0.04			0.03
<90 days	2818 (88.49%)	11,247 (89.29%)		2239 (89.59%)	2217 (88.71%)	
90–365 days	182 (5.78%)	612 (4.86%)		143 (5.73%)	127 (5.09%)	
>365 days	149 (4.73%)	737 (5.85%)		117 (4.67%)	155 (6.20%)	
Lipophilic statin (%)			0.02			0.01
<90 days	2864 (90.95%)	11,391 (90.43%)		2278 (91.03%)	2247 (89.92%)	
90–365 days	162 (5.14%)	586 (4.65%)		128 (5.11%)	122 (4.87%)	
>365 days	123 (3.91%)	619 (4·91%)		96 (3.86%)	130 (5.21%)	
Hydrophilic statin (%)			0.00			0.01
<90 days	3092 (98.19%)	12,379 (98.28%)		2455 (98.23%)	2454 (98.20%)	
90–365 days	33 (1.05%)	133 (1.06%)		26 (1.02%)	27 (1.09%)	
>365 days	24 (10.76%)	84 (0.67%)		19 (0.75%)	18 (0.71%)	

Abbreviations: RA, rheumatoid arthritis; SMD, standardized mean difference; SBP, systolic blood pressure; DBP, diastolic blood pressure; SD, standard deviation; FBG, fasting blood glucose; CCI, Charlson Comorbidity Index. † Obesity (BMI, body mass index, kg/m^2^) was categorized as <18.5 (underweight), ≥18.5 to <23 (normal), ≥23 to <25 (overweight), ≥25 to <30 (obese I), and ≥30 (obese II).

**Table 2:** Crude and adjusted odds ratios of statin types and use duration for RA.

Characteristics	No. of RA	No. of Control	OR for RA (95% CI)			
	Exposure/Total (%)	Exposure/Total (%)	Crude	*p* Value	Overlap Weighted Model †	*p* Value
Any statin						
<90 days	2818/3149 (89.5%)	11,247/12,596 (89.3%)	1		1	
90–365 days	182/3149 (5.8%)	612/12,596 (4.9%)	1.19 (1.00–1.41)	0.049 *	1.11 (0.96–1.28)	0.173
>365 days	149/3149 (4.7%)	737/12,596 (5.9%)	0.81 (0.67–0.97)	0.020 *	0.73 (0.63–0.85)	<0.001 *
Lipophilic statin						
<90 days	2864/3149 (90.9%)	11,391/12,596 (90.4%)	1		1	
90–365 days	162/3149 (5.1%)	586/12,596 (4.7%)	1.10 (0.92–1.31)	0.298	1.02 (0.88–1.19)	0.774
>365 days	123/3149 (3.9%)	619/12,596 (4.9%)	0.79 (0.65–0.96)	0.020 *	0.71 (0.61–0.84)	<0.001 *
Hydrophilic statin						
<90 days	3092/3149 (98.2%)	12,379/12,596 (98.3%)	1		1	
90–365 days	33/3149 (1.0%)	133/12,596 (1.1%)	0.99 (0.68–1.46)	0.973	0.94 (0.69–1.28)	0.695
>365 days	24/3149 (0.8%)	84/12,596 (0.7%)	1.14 (0.73–1.80)	0.563	1.05 (0.73–1.52)	0.788

Abbreviations: No., number; RA, rheumatoid arthritis; OR, odds ratio; CI, confidence interval. * Significance at *p* < 0.05. † Adjusted for age, sex, income, region of residence, systolic blood pressure, diastolic blood pressure, fasting blood glucose, total cholesterol, hemoglobin, obesity, smoking, alcohol consumption, dyslipidemia history, and Charlson Comorbidity Index scores.

## Data Availability

All data are available from the database of the National Health Insurance Sharing Service (NHISS) https://nhiss.nhis.or.kr/ (last accessed on 1 January 2020). The NHISS allows access to all of these data for the any researcher who promises to follow the research ethics at some processing charge. If you want to access the data of this article, you can download them from the website after promising to follow the research ethics.

## Supplementary Materials

The following supporting information can be downloaded at https://www.mdpi.com/article/10.3390/jpm12040559/s1, Figure S1: Forest plots depicting the association between use duration of hydrophilic statin and a subsequent risk of incident rheumatoid arthritis (RA) in each subgroup; Table S1: Crude and overlap propensity score weighted odds ratios of dates of any statin prescription for RA; Table S2: Crude and overlap propensity score weighted odds ratios of dates of lipophilic statin prescription for RA; Table S3: Crude and overlap propensity score weighted odds ratios of dates of hydrophilic statin prescription for RA.
