# Supplementary material for: Incident Rheumatoid Arthritis Following Statin Use: From the View of a National Cohort Study in Korea
Source: J Pers Med. 2022 Apr 1;12(4):559. doi: 10.3390/jpm12040559 (PMC9032630; doi:10.3390/jpm12040559)
Supplement: Supplementary file 1 [file jpm-12-00559-s001.zip › jpm-1607747-supplementary.PDF]

Table S1. Crude and overlap propensity score weighted odd ratios of dates of any statin prescription for RA.

Characteristics	N of RA (exposure/total, %)	N of Control (exposure/total, %)	Odd ratios for RA (95% confidence interval) Crude	P-value	Overlap weighted model †	P-value
Age < 60 years old (n=8,735)						
< 90 days	1,632/1,747 (93.4%)	6,552/6,988 (93.8%)	1		1	
90 - 365 days	73/1,747 (4.2%)	226/6,988 (3.2%)	1.30 (0.99-1.70)	0.059	1.17 (0.93–1.47)	0.176
> 365 days	42/1,747 (2.4%)	210/6,988 (3.0%)	0.80 (0.57-1.12)	0.200	0.71 (0.54–0.92)	0.010*
Age ≥ 60 years old (n=7,010)						
< 90 days	1,186/1,402 (84.6%)	4,695/5,608 (83.7%)	1		1	
90 - 365 days	109/1,402 (7.8%)	386/5,608 (6.9%)	1.12 (0.90-1.40)	0.325	1.10 (0.91–1.32)	0.341
> 365 days	107/1,402 (7.6%)	527/5,608 (9.4%)	0.80 (0.65-1.00)	0.049*	0.77 (0.64–0.92)	0.004*
Male (n=4,225)						
< 90 days	770/845 (91.1%)	3,028/3,380 (89.6%)	1		1	
90 - 365 days	35/845 (4.1%)	134/3,380 (4.0%)	1.03 (0.70-1.50)	0.890	0.90 (0.66–1.24)	0.517
> 365 days	40/845 (4.7%)	218/3,380 (6.4%)	0.72 (0.51-1.02)	0.065	0.62 (0.47–0.82)	0.001*
Female (n=11,520)						
< 90 days	2,048/2,304 (88.9%)	8,219/9,216 (89.2%)	1		1	
90 - 365 days	147/2,304 (6.4%)	478/9,216 (5.2%)	1.23 (1.02-1.49)	0.031*	1.16 (0.99–1.37)	0.068
> 365 days	109/2,304 (4.7%)	519/9,216 (5.6%)	0.84 (0.68-1.04)	0.114	0.76 (0.64–0.91)	0.002*
Low income groups (n=7,730)						
< 90 days	1,398/1,546 (90.4%)	5,588/6,184 (90.4%)	1		1	
90 - 365 days	89/1,546 (5.8%)	284/6,184 (4.6%)	1.25 (0.98-1.60)	0.072	1.12 (0.91–1.38)	0.271
> 365 days	59/1,546 (3.8%)	312/6,184 (5.0%)	0.76 (0.57-1.00)	0.054	0.69 (0.55–0.87)	0.002*
High income groups (n=8,015)						
< 90 days	1,420/1,603 (88.6%)	5,659/6,412 (88.3%)	1		1	
90 - 365 days	93/1,603 (5.8%)	328/6,412 (5.1%)	1.13 (0.89-1.43)	0.313	1.10 (0.90–1.34)	0.369
> 365 days	90/1,603 (5.6%)	425/6,412 (6.6%)	0.84 (0.67-1.07)	0.157	0.75 (0.61–0.91)	0.003*
Urban residents (n=6,800)						
< 90 days	1,210/1,360 (89.0%)	4,793/5,440 (88.1%)	1		1	
90 - 365 days	81/1,360 (6.0%)	298/5,440 (5.5%)	1.08 (0.84-1.39)	0.568	1.05 (0.85–1.30)	0.650
> 365 days	69/1,360 (5.1%)	349/5,440 (6.4%)	0.78 (0.60-1.02)	0.072	0.73 (0.58–0.90)	0.004*
Rural residents (n=8,945)						
< 90 days	1,608/1,789 (89.9%)	6,454/7,156 (90.2%)	1		1	
90 - 365 days	101/1,789 (5.6%)	314/7,156 (4.4%)	1.29 (1.03-1.63)	0.030*	1.16 (0.95–1.41)	0.136
> 365 days	80/1,789 (4.5%)	388/7,156 (5.4%)	0.83 (0.65-1.06)	0.133	0.73 (0.60–0.90)	0.003*
Underweight (n=344)						
< 90 days	60/61 (98.4%)	269/283 (95.1%)	1		1	
90 - 365 days	1/61 (1.6%)	6/283 (2.1%)	N/A		N/A	
> 365 days	0/61 (0.0%)	8/283 (2.8%)	N/A		N/A	
Normal weight (n=5,768)						
< 90 days	1,120/1,201 (93.3%)	4,214/4,567 (92.3%)	1		1	
90 - 365 days	46/1,201 (3.8%)	161/4,567 (3.5%)	1.08 (0.77-1.50)	0.672	0.93 (0.70–1.23)	0.616
> 365 days	35/1,201 (2.9%)	192/4,567 (4.2%)	0.69 (0.48-0.99)	0.044*	0.57 (0.43–0.77)	<0.001*
Overweight (n=4,186)						
< 90 days	742/826 (89.8%)	2,980/3,360 (88.7%)	1		1	

90 - 365 days	44/826 (5.3%)	165/3,360 (4.9%)	1.07 (0.76-1.51)	0.694	0.92 (0.69–1.22)	0.569
> 365 days	40/826 (4.8%)	215/3,360 (6.4%)	0.75 (0.53-1.06)	0.100	0.65 (0.50–0.86)	0.003*
Obese (n=5,447)						
< 90 days	896/1,061 (84.4%)	3,784/4,386 (86.3%)	1		1	
90 - 365 days	91/1,061 (8.6%)	280/4,386 (6.4%)	1.37 (1.07-1.76)	0.012*	1.38 (1.12–1.71)	0.003*
> 365 days	74/1,061 (7.0%)	322/4,386 (7.3%)	0.97 (0.75-1.26)	0.824	0.93 (0.75–1.16)	0.513
Non-smoker (n=13,079)						
< 90 days	2,311/2,585 (89.4%)	9,385/10,494 (89.4%)	1		1	
90 - 365 days	155/2,585 (6.0%)	520/10,494 (5.0%)	1.21 (1.01-1.46)	0.043*	1.15 (0.98–1.34)	0.086
> 365 days	119/2,585 (4.6%)	589/10,494 (5.6%)	0.82 (0.67-1.00)	0.055	0.75 (0.64–0.88)	0.001*
Past and current smoker (n=2,666)						
< 90 days	507/564 (89.9%)	1,862/2,102 (88.6%)	1		1	
90 - 365 days	27/564 (4.8%)	92/2,102 (4.4%)	1.08 (0.69-1.67)	0.739	0.89 (0.61–1.30)	0.559
> 365 days	30/564 (5.3%)	148/2,102 (7.0%)	0.74 (0.50-1.12)	0.153	0.63 (0.45–0.88)	0.007*
Alcohol consumption <1 time a week (n=12,420)						
< 90 days	2,282/2,537 (89.9%)	8,871/9,883 (89.8%)	1		1	
90 - 365 days	142/2,537 (5.6%)	485/9,883 (4.9%)	1.14 (0.94-1.38)	0.188	1.09 (0.93–1.28)	0.302
> 365 days	113/2,537 (4.5%)	527/9,883 (5.3%)	0.83 (0.68-1.03)	0.087	0.78 (0.66–0.92)	0.004*
Alcohol consumption ≥1 time a week (n=3,325)						
< 90 days	536/612 (87.6%)	2,376/2,713 (87.6%)	1		1	
90 - 365 days	40/612 (6.5%)	127/2,713 (4.7%)	1.40 (0.97-2.02)	0.075	1.10 (0.80–1.51)	0.545
> 365 days	36/612 (5.9%)	210/2,713 (7.7%)	0.76 (0.53-1.10)	0.142	0.56 (0.41–0.75)	<0.001*
SBP < 140 mmHg and DBP < 90 mmHg (n=11,670)						
< 90 days	2,152/2,403 (89.6%)	8,293/9,267 (89.5%)	1		1	
90 - 365 days	138/2,403 (5.7%)	449/9,267 (4.8%)	1.18 (0.97-1.44)	0.092	1.07 (0.90–1.26)	0.451
> 365 days	113/2,403 (4.7%)	525/9,267 (5.7%)	0.83 (0.67-1.02)	0.079	0.73 (0.61–0.87)	<0.001*
SBP ≥ 140 mmHg or DBP ≥ 90 mmHg (n=4,075)						
< 90 days	666/746 (89.3%)	2,954/3,329 (88.7%)	1		1	
90 - 365 days	44/746 (5.9%)	163/3,329 (4.9%)	1.20 (0.85-1.69)	0.304	1.18 (0.89–1.56)	0.249
> 365 days	36/746 (4.8%)	212/3,329 (6.4%)	0.75 (0.52-1.08)	0.126	0.72 (0.54–0.95)	0.021*
Fasting blood glucose < 100 mg/dL (n=10,584)						
< 90 days	2,028/2,200 (92.2%)	7,692/8,384 (91.7%)	1		1	
90 - 365 days	91/2,200 (4.1%)	337/8,384 (4.0%)	1.02 (0.81-1.30)	0.843	0.92 (0.75–1.12)	0.390
> 365 days	81/2,200 (3.7%)	355/8,384 (4.2%)	0.87 (0.68-1.11)	0.250	0.75 (0.61–0.92)	0.005*
Fasting blood glucose ≥ 100 mg/dL (n=5,161)						
< 90 days	790/949 (83.2%)	3,555/4,212 (84.4%)	1		1	
90 - 365 days	91/949 (9.6%)	275/4,212 (6.5%)	1.49 (1.16-1.91)	0.002*	1.39 (1.12–1.72)	0.002*
> 365 days	68/949 (7.2%)	382/4,212 (9.1%)	0.80 (0.61-1.05)	0.107	0.74 (0.60–0.92)	0.006*
Total cholesterol < 200 mg/dL (n=7,977)						
< 90 days	1,489/1,640 (90.8%)	5,689/6,337 (89.8%)	1		1	
90 - 365 days	60/1,640 (3.7%)	190/6,337 (3.0%)	1.21 (0.90-1.62)	0.213	1.01 (0.78–1.29)	0.966
> 365 days	91/1,640 (5.5%)	458/6,337 (7.2%)	0.76 (0.60-0.96)	0.020*	0.66 (0.54–0.80)	<0.001*
Total cholesterol ≥ 200 mg/dL (n=7,768)						
< 90 days	1,329/1,509 (88.1%)	5,558/6,259 (88.8%)	1		1	
90 - 365 days	122/1,509 (8.1%)	422/6,259 (6.7%)	1.21 (0.98-1.49)	0.077	1.16 (0.97–1.38)	0.099
> 365 days	58/1,509 (3.8%)	279/6,259 (4.5%)	0.87 (0.65-1.16)	0.343	0.84 (0.67–1.06)	0.137

Hemoglobin ≥12 g/dL for men and > 10 g/dL for women (n=301)						
< 90 days	70/75 (93.3%)	208/226 (92.0%)	1		1	
90 - 365 days	3/75 (4.0%)	6/226 (2.7%)	1.49 (0.36-6.10)	0.583	1.13 (0.28–4.51)	0.865
> 365 days	2/75 (2.7%)	12/226 (5.3%)	0.50 (0.11-2.27)	0.365	0.45 (0.12–1.69)	0.237
Hemoglobin < 12 g/dL for men and < 10 g/dL for women (n=15,444)						
< 90 days	2,748/3,074 (89.4%)	11,039/12,370 (89.2%)	1		1	
90 - 365 days	179/3,074 (5.8%)	606/12,370 (4.9%)	1.19 (1.00-1.41)	0.051	1.10 (0.95–1.27)	0.197
> 365 days	147/3,074 (4.8%)	725/12,370 (5.9%)	0.81 (0.68-0.98)	0.027*	0.74 (0.63–0.85)	<0.001*
CCI scores = 0 (n=11,002)						
< 90 days	1,869/2,058 (90.8%)	8,100/8,944 (90.6%)	1		1	
90 - 365 days	105/2,058 (5.1%)	398/8,944 (4.4%)	1.14 (0.92-1.43)	0.234	1.07 (0.90–1.29)	0.436
> 365 days	84/2,058 (4.1%)	446/8,944 (5.0%)	0.82 (0.64-1.04)	0.095	0.76 (0.63–0.92)	0.004*
CCI scores = 1 (n=2,213)						
< 90 days	501/573 (87.4%)	1,423/1,640 (86.8%)	1		1	
90 - 365 days	39/573 (6.8%)	95/1,640 (5.8%)	1.17 (0.79-1.72)	0.436	1.22 (0.85–1.76)	0.273
> 365 days	33/573 (5.8%)	122/1,640 (7.4%)	0.77 (0.52-1.14)	0.194	0.79 (0.55–1.13)	0.205
CCI scores ≥ 2 (n=2,530)						
< 90 days	448/518 (86.5%)	1,724/2,012 (85.7%)	1		1	
90 - 365 days	38/518 (7.3%)	119/2,012 (5.9%)	1.23 (0.84-1.80)	0.287	1.09 (0.78–1.52)	0.601
> 365 days	32/518 (6.2%)	169/2,012 (8.4%)	0.73 (0.49-1.08)	0.114	0.62 (0.45–0.86)	0.004*
Non dyslipidemia history (n=8,594)						
< 90 days	1,564/1,596 (98.0%)	6,885/6,998 (98.4%)	1		1	
90 - 365 days	16/1,596 (1.0%)	54/6,998 (0.8%)	1.30 (0.74-2.29)	0.353	1.34 (0.84–2.15)	0.218
> 365 days	16/1,596 (1.0%)	59/6,998 (0.8%)	1.19 (0.69-2.08)	0.532	1.18 (0.75–1.86)	0.474
Dyslipidemia history (n=7,151)						
< 90 days	1,254/1,553 (80.7%)	4,362/5,598 (77.9%)	1		1	
90 - 365 days	166/1,553 (10.7%)	558/5,598 (10.0%)	1.03 (0.86-1.24)	0.716	1.12 (0.95–1.31)	0.169
> 365 days	133/1,553 (8.6%)	678/5,598 (12.1%)	0.68 (0.56-0.83)	<0.001*	0.72 (0.61–0.84)	<0.001*

Abbreviations: RA, Rheumatoid arthritis; CCI, Charlson Comorbidity Index; SBP, Systolic blood pressure; DBP, Diastolic blood pressure;

* Significance at P < 0.05

† Adjusted for age, sex, income, region of residence, SBP, DBP, fasting blood glucose, total cholesterol, hemoglobin, obesity, smoking, alcohol consumption, dyslipidemia history, and CCI scores.

Table S2. Crude and overlap propensity score weighted odd ratios of dates of lipophilic statin prescription for RA

Characteristics	N of RA (exposure/total, %)	N of Control (exposure/total, %)	Odd ratios for RA (95% confidence interval)			
			Crude	P-value	Overlap weighted model †	P-value
Age < 60 years old (n=8,735)						
< 90 days	1,650/1,747 (94.4%)	6,597/6,988 (94.4%)	1		1	
90 - 365 days	67/1,747 (3.8%)	209/6,988 (3.0%)	1.28 (0.97-1.70)	0.083	1.16 (0.91–1.47)	0.229
> 365 days	30/1,747 (1.7%)	182/6,988 (2.6%)	0.66 (0.45-0.97)	0.037*	0.58 (0.43–0.78)	<0.001*
Age ≥ 60 years old (n=7,010)						
< 90 days	1,214/1,402 (86.6%)	4,794/5,608 (85.5%)	1		1	
90 - 365 days	95/1,402 (6.8%)	377/5,608 (6.7%)	1.00 (0.79-1.26)	0.967	0.97 (0.80–1.18)	0.775
> 365 days	93/1,402 (6.6%)	437/5,608 (7.8%)	0.84 (0.67-1.06)	0.143	0.80 (0.66–0.97)	0.026*
Male (n= 4,225)						
< 90 days	784/845 (92.8%)	3,075/3,380 (91.0%)	1		1	
90 - 365 days	29/845 (3.4%)	131/3,380 (3.9%)	0.87 (0.58-1.31)	0.499	0.77 (0.55–1.07)	0.119
> 365 days	32/845 (3.8%)	174/3,380 (5.1%)	0.72 (0.49-1.06)	0.097	0.62 (0.46–0.85)	0.003*
Female (n=11,520)						
< 90 days	2,080/2,304 (90.3%)	8,316/9,216 (90.2%)	1		1	
90 - 365 days	133/2,304 (5.8%)	455/9,216 (4.9%)	1.17 (0.96-1.43)	0.125	1.10 (0.93–1.30)	0.283
> 365 days	91/2,304 (3.9%)	445/9,216 (4.8%)	0.82 (0.65-1.03)	0.087	0.74 (0.61–0.89)	0.001*
Low income groups (n=7,730)						
< 90 days	1,414/1,546 (91.5%)	5,642/6,184 (91.2%)	1		1	
90 - 365 days	83/1,546 (5.4%)	272/6,184 (4.4%)	1.22 (0.95-1.57)	0.127	1.10 (0.89–1.36)	0.386
> 365 days	49/1,546 (3.2%)	270/6,184 (4.4%)	0.72 (0.53-0.99)	0.041*	0.67 (0.52–0.86)	0.001*
High income groups (n=8,015)						
< 90 days	1,450/1,603 (90.5%)	5,749/6,412 (89.7%)	1		1	
90 - 365 days	79/1,603 (4.9%)	314/6,412 (4.9%)	1.00 (0.77-1.29)	0.985	0.96 (0.78–1.18)	0.686
> 365 days	74/1,603 (4.6%)	349/6,412 (5.4%)	0.84 (0.65-1.09)	0.186	0.74 (0.60–0.91)	0.005*
Urban residents (n=6,800)						
< 90 days	1,229/1,360 (90.4%)	4,879/5,440 (89.7%)	1		1	
90 - 365 days	74/1,360 (5.4%)	274/5,440 (5.0%)	1.07 (0.82-1.40)	0.605	1.05 (0.84–1.31)	0.677
> 365 days	57/1,360 (4.2%)	287/5,440 (5.3%)	0.79 (0.59-1.05)	0.110	0.74 (0.58–0.94)	0.012*
Rural residents (n=8,945)						
< 90 days	1,635/1,789 (91.4%)	6,512/7,156 (91.0%)	1		1	
90 - 365 days	88/1,789 (4.9%)	312/7,156 (4.4%)	1.12 (0.88-1.43)	0.348	1.00 (0.82–1.23)	0.983
> 365 days	66/1,789 (3.7%)	332/7,156 (4.6%)	0.79 (0.60-1.04)	0.090	0.70 (0.56–0.86)	0.001*
Underweight (n=344)						
< 90 days	60/61 (98.4%)	273/283 (96.5%)	1		1	
90 - 365 days	1/61 (1.6%)	4/283 (1.4%)	N/A		N/A	
> 365 days	0/61 (0.0%)	6/283 (2.1%)	N/A		N/A	
Normal weight (n=5,768)						
< 90 days	1,133/1,201 (94.3%)	4,241/4,567 (92.9%)	1		1	
90 - 365 days	41/1,201 (3.4%)	163/4,567 (3.6%)	0.94 (0.66-1.33)	0.735	0.81 (0.61–1.08)	0.152
> 365 days	27/1,201 (2.2%)	163/4,567 (3.6%)	0.62 (0.41-0.94)	0.023*	0.51 (0.37–0.71)	<0.001*
Overweight (n=4,186)						
< 90 days	756/826 (91.5%)	3,031/3,360 (90.2%)	1		1	

90 - 365 days	36/826 (4.4%)	149/3,360 (4.4%)	0.97 (0.67-1.41)	0.867	0.84 (0.62–1.14)	0.270
> 365 days	34/826 (4.1%)	180/3,360 (5.4%)	0.76 (0.52-1.10)	0.147	0.67 (0.50–0.90)	0.009*
Obese (n=5,447)						
< 90 days	915/1,061 (86.2%)	3,846/4,386 (87.7%)	1		1	
90 - 365 days	84/1,061 (7.9%)	270/4,386 (6.2%)	1.31 (1.01-1.69)	0.039*	1.31 (1.06–1.63)	0.014*
> 365 days	62/1,061 (5.8%)	270/4,386 (6.2%)	0.97 (0.73-1.28)	0.808	0.92 (0.73–1.17)	0.502
Non-smoker (n=13,079)						
< 90 days	2,345/2,585 (90.7%)	9,504/10,494 (90.6%)	1		1	
90 - 365 days	140/2,585 (5.4%)	494/10,494 (4.7%)	1.15 (0.95-1.39)	0.160	1.09 (0.92–1.28)	0.314
> 365 days	100/2,585 (3.9%)	496/10,494 (4.7%)	0.82 (0.66-1.02)	0.071	0.75 (0.63–0.89)	0.001*
Past and current smoker (n=2,666)						
< 90 days	519/564 (92.0%)	1,887/2,102 (89.8%)	1		1	
90 - 365 days	22/564 (3.9%)	92/2,102 (4.4%)	0.87 (0.54-1.40)	0.564	0.72 (0.48–1.06)	0.098
> 365 days	23/564 (4.1%)	123/2,102 (5.9%)	0.68 (0.43-1.07)	0.097	0.57 (0.39–0.83)	0.004*
Alcohol consumption <1 time a week (n=12,420)						
< 90 days	2,315/2,537 (91.2%)	8,982/9,883 (90.9%)	1		1	
90 - 365 days	126/2,537 (5.0%)	456/9,883 (4.6%)	1.07 (0.88-1.31)	0.501	1.03 (0.87–1.22)	0.755
> 365 days	96/2,537 (3.8%)	445/9,883 (4.5%)	0.84 (0.67-1.05)	0.122	0.78 (0.65–0.94)	0.009*
Alcohol consumption ≥1 time a week (n=3,325)						
< 90 days	549/612 (89.7%)	2,409/2,713 (88.8%)	1		1	
90 - 365 days	36/612 (5.9%)	130/2,713 (4.8%)	1.22 (0.83-1.78)	0.316	0.93 (0.68–1.29)	0.675
> 365 days	27/612 (4.4%)	174/2,713 (6.4%)	0.68 (0.45-1.03)	0.071	0.49 (0.35–0.68)	<0.001*
SBP < 140 mmHg and DBP < 90 mmHg (n=11,670)						
< 90 days	2,186/2,403 (91.0%)	8,397/9,267 (90.6%)	1		1	
90 - 365 days	124/2,403 (5.2%)	431/9,267 (4.7%)	1.11 (0.90-1.36)	0.340	1.00 (0.84–1.19)	0.965
> 365 days	93/2,403 (3.9%)	439/9,267 (4.7%)	0.81 (0.65-1.02)	0.077	0.72 (0.59–0.86)	0.001*
SBP ≥ 140 mmHg or DBP ≥ 90 mmHg (n=4,075)						
< 90 days	678/746 (90.9%)	2,994/3,329 (89.9%)	1		1	
90 - 365 days	38/746 (5.1%)	155/3,329 (4.7%)	1.08 (0.75-1.56)	0.670	1.07 (0.80–1.43)	0.660
> 365 days	30/746 (4.0%)	180/3,329 (5.4%)	0.74 (0.50-1.09)	0.129	0.69 (0.51–0.93)	0.017*
Fasting blood glucose < 100 mg/dL (n=10,584)						
< 90 days	2,054/2,200 (93.4%)	7,763/8,384 (92.6%)	1		1	
90 - 365 days	80/2,200 (3.6%)	316/8,384 (3.8%)	0.96 (0.75-1.23)	0.729	0.85 (0.69–1.05)	0.125
> 365 days	66/2,200 (3.0%)	305/8,384 (3.6%)	0.82 (0.62-1.07)	0.145	0.71 (0.57–0.88)	0.002*
Fasting blood glucose ≥ 100 mg/dL (n=5,161)						
< 90 days	810/949 (85.4%)	3,628/4,212 (86.1%)	1		1	
90 - 365 days	82/949 (8.6%)	270/4,212 (6.4%)	1.36 (1.05-1.76)	0.020*	1.27 (1.02–1.57)	0.033*
> 365 days	57/949 (6.0%)	314/4,212 (7.5%)	0.81 (0.61-1.09)	0.165	0.75 (0.60–0.95)	0.018*
Total cholesterol < 200 mg/dL (n=7,977)						
< 90 days	1,505/1,640 (91.8%)	5,758/6,337 (90.9%)	1		1	
90 - 365 days	56/1,640 (3.4%)	189/6,337 (3.0%)	1.13 (0.84-1.54)	0.418	0.95 (0.74–1.23)	0.699
> 365 days	79/1,640 (4.8%)	390/6,337 (6.2%)	0.78 (0.60-0.99)	0.044*	0.68 (0.55–0.84)	<0.001*
Total cholesterol ≥ 200 mg/dL (n=7,768)						
< 90 days	1,359/1,509 (90.1%)	5,633/6,259 (90.0%)	1		1	
90 - 365 days	106/1,509 (7.0%)	397/6,259 (6.3%)	1.11 (0.89-1.38)	0.371	1.06 (0.88–1.27)	0.563
> 365 days	44/1,509 (2.9%)	229/6,259 (3.7%)	0.80 (0.57-1.11)	0.174	0.76 (0.59–0.98)	0.034*

Hemoglobin ≥ 12 g/dL for men and > 10 g/dL for women (n=301)						
< 90 days	71/75 (94.7%)	210/226 (92.9%)	1		1	
90 - 365 days	3/75 (4.0%)	8/226 (3.5%)	1.11 (0.29-4.30)	0.881	0.88 (0.42–3.21)	0.844
> 365 days	1/75 (1.3%)	8/226 (3.5%)	0.37 (0.05-3.01)	0.352	0.27 (0.04–1.61)	0.149
Hemoglobin < 12 g/dL for men and < 10 g/dL for women (n=15,444)						
< 90 days	2,793/3,074 (90.9%)	11,181/12,370 (90.4%)	1		1	
90 - 365 days	159/3,074 (5.2%)	578/12,370 (4.7%)	1.10 (0.92-1.32)	0.295	1.02 (0.88–1.19)	0.785
> 365 days	122/3,074 (4.0%)	611/12,370 (4.9%)	0.80 (0.66-0.98)	0.027*	0.72 (0.61–0.84)	<0.001*
CCI scores = 0 (n=11,002)						
< 90 days	1,893/2,058 (92.0%)	8,185/8,944 (91.5%)	1		1	
90 - 365 days	98/2,058 (4.8%)	376/8,944 (4.2%)	1.13 (0.90-1.42)	0.304	1.05 (0.88–1.27)	0.579
> 365 days	67/2,058 (3.3%)	383/8,944 (4.3%)	0.76 (0.58-0.99)	0.039*	0.70 (0.57–0.86)	0.001*
CCI scores = 1 (n=2,213)						
< 90 days	512/573 (89.4%)	1,442/1,640 (87.9%)	1		1	
90 - 365 days	33/573 (5.8%)	93/1,640 (5.7%)	1.00 (0.66-1.51)	0.998	1.04 (0.71–1.51)	0.850
> 365 days	28/573 (4.9%)	105/1,640 (6.4%)	0.75 (0.49-1.15)	0.191	0.78 (0.53–1.14)	0.194
CCI scores ≥ 2 (n=2,530)						
< 90 days	459/518 (88.6%)	1,764/2,012 (87.7%)	1		1	
90 - 365 days	31/518 (6.0%)	117/2,012 (5.8%)	1.02 (0.68-1.53)	0.931	0.92 (0.65–1.31)	0.657
> 365 days	28/518 (5.4%)	131/2,012 (6.5%)	0.82 (0.54-1.25)	0.360	0.70 (0.49–1.00)	0.048*
Non dyslipidemia history (n=8,594)						
< 90 days	1,571/1,596 (98.4%)	6,898/6,998 (98.6%)	1		1	
90 - 365 days	13/1,596 (0.8%)	51/6,998 (0.7%)	1.12 (0.61-2.06)	0.718	1.17 (0.71–1.92)	0.548
> 365 days	12/1,596 (0.8%)	49/6,998 (0.7%)	1.08 (0.57-2.03)	0.822	1.07 (0.64–1.79)	0.800
Dyslipidemia history (n=7,151)						
< 90 days	1,293/1,553 (83.3%)	4,493/5,598 (80.3%)	1		1	
90 - 365 days	149/1,553 (9.6%)	535/5,598 (9.6%)	0.97 (0.80-1.17)	0.738	1.04 (0.88–1.22)	0.636
> 365 days	111/1,553 (7.1%)	570/5,598 (10.2%)	0.68 (0.55-0.84)	<0.001*	0.71 (0.60–0.85)	<0.001*

Abbreviations: RA, Rheumatoid arthritis; CCI, Charlson Comorbidity Index; SBP, Systolic blood pressure; DBP, Diastolic blood pressure;

* Significance at P < 0.05

† Adjusted for age, sex, income, region of residence, SBP, DBP, fasting blood glucose, total cholesterol, hemoglobin, obesity, smoking, alcohol consumption, dyslipidemia history, and CCI scores.

Table S3. Crude and overlap propensity score weighted odd ratios of dates of hydrophilic statin prescription for RA

Characteristics	N of RA (exposure/total, %)	N of Control (exposure/total, %)	Odd ratios for RA (95% confidence interval) Crude	P-value	Overlap weighted model †	P-value
Age < 60 years old (n=8,735)						
< 90 days	1,727/1,747 (98.9%)	6,930/6,988 (99.2%)	1		1	
90 - 365 days	0/1,747 (0.5%)	37/6,988 (0.5%)	0.98 (0.47-2.03)	0.948	0.87 (0.48–1.55)	0.629
> 365 days	11/1,747 (0.6%)	21/6,988 (0.3%)	2.10 (1.01-4.37)	0.047*	1.85 (0.97–3.52)	0.061
Age ≥ 60 years old (n=7,010)						
< 90 days	1,365/1,402 (97.4%)	5,449/5,608 (97.2%)	1		1	
90 - 365 days	24/1,402 (1.7%)	96/5,608 (1.7%)	1.00 (0.64-1.57)	0.993	0.99 (0.69–1.43)	0.961
> 365 days	13/1,402 (0.9%)	63/5,608 (1.1%)	0.82 (0.45-1.50)	0.527	0.80 (0.50–1.28)	0.355
Male (n=4,225)						
< 90 days	830/845 (98.2%)	3,312/3,380 (98.0%)	1		1	
90 - 365 days	8/845 (0.9%)	29/3,380 (0.9%)	1.10 (0.50-2.42)	0.811	0.98 (0.52–1.87)	0.957
> 365 days	7/845 (0.8%)	39/3,380 (1.2%)	0.72 (0.32-1.61)	0.419	0.65 (0.36–1.19)	0.162
Female (n=11,520)						
< 90 days	2,262/2,304 (98.2%)	9,067/9,216 (98.4%)	1		1	
90 - 365 days	25/2,304 (1.1%)	104/9,216 (1.1%)	0.96 (0.62-1.49)	0.868	0.92 (0.65–1.31)	0.636
> 365 days	17/2,304 (0.7%)	45/9,216 (0.5%)	1.52 (0.87-2.65)	0.146	1.41 (0.88–2.28)	0.157
Low income groups (n=7,730)						
< 90 days	1,521/1,546 (98.4%)	6,103/6,184 (98.7%)	1		1	
90 - 365 days	16/1,546 (1.0%)	53/6,184 (0.9%)	1.21 (0.69-2.12)	0.504	1.11 (0.70–1.76)	0.665
> 365 days	9/1,546 (0.6%)	28/6,184 (0.5%)	1.29 (0.61-2.74)	0.508	1.14 (0.62–2.11)	0.664
High income groups (n=8,015)						
< 90 days	1,571/1,603 (98.0%)	6,276/6,412 (97.9%)	1		1	
90 - 365 days	17/1,603 (1.1%)	80/6,412 (1.2%)	0.85 (0.50-1.44)	0.542	0.81 (0.53–1.23)	0.323
> 365 days	15/1,603 (0.9%)	56/6,412 (0.9%)	1.07 (0.60-1.90)	0.817	0.98 (0.62–1.56)	0.946
Urban residents (n=6,800)						
< 90 days	1,333/1,360 (98.0%)	5,323/5,440 (97.8%)	1		1	
90 - 365 days	17/1,360 (1.3%)	68/5,440 (1.3%)	1.00 (0.58-1.70)	0.995	0.99 (0.64–1.53)	0.950
> 365 days	10/1,360 (0.7%)	49/5,440 (0.9%)	0.81 (0.41-1.61)	0.557	0.76 (0.45–1.28)	0.302
Rural residents (n=8,945)						
< 90 days	1,759/1,789 (98.3%)	7,056/7,156 (98.6%)	1		1	
90 - 365 days	16/1,789 (0.9%)	65/7,156 (0.9%)	0.99 (0.57-1.71)	0.964	0.92 (0.60–1.43)	0.715
> 365 days	14/1,789 (0.8%)	35/7,156 (0.5%)	1.61 (0.86-2.99)	0.135	1.45 (0.86–2.46)	0.164
Underweight (n=344)						
< 90 days	61/61 (100.0%)	279/283 (98.6%)	1		1	
90 - 365 days	0/61 (0.0%)	2/283 (0.7%)	N/A		N/A	
> 365 days	0/61 (0.0%)	2/283 (0.7%)	N/A		N/A	
Normal weight (n=5,768)						
< 90 days	1,183/1,201 (98.5%)	4,520/4,567 (99.0%)	1		1	
90 - 365 days	12/1,201 (1.0%)	28/4,567 (0.6%)	1.64 (0.83-3.23)	0.154	1.45 (0.80–2.62)	0.224
> 365 days	6/1,201 (0.5%)	19/4,567 (0.4%)	1.21 (0.48-3.03)	0.689	1.11 (0.52–2.38)	0.789
Overweight (n=4,186)						
< 90 days	811/826 (98.2%)	3,295/3,360 (98.1%)	1		1	

90 - 365 days	9/826 (1.1%)	39/3,360 (1.2%)	0.94 (0.45-1.95)	0.865	0.86 (0.48–1.54)	0.613
> 365 days	6/826 (0.7%)	26/3,360 (0.8%)	0.94 (0.39-2.29)	0.889	0.82 (0.41–1.64)	0.574
Obese (n=5,447)						
< 90 days	1,037/1,061 (97.7%)	4,285/4,386 (97.7%)	1		1	
90 - 365 days	12/1,061 (1.1%)	64/4,386 (1.5%)	0.77 (0.42-1.44)	0.420	0.77 (0.48–1.24)	0.278
> 365 days	12/1,061 (1.1%)	37/4,386 (0.8%)	1.34 (0.70-2.58)	0.380	1.29 (0.75–2.20)	0.357
Non-smoker (n=13,079)						
< 90 days	2,542/2,585 (98.3%)	10,317/10,494 (98.3%)	1		1	
90 - 365 days	25/2,585 (1.0%)	114/10,494 (1.1%)	0.89 (0.58-1.38)	0.600	0.85 (0.60–1.19)	0.343
> 365 days	18/2,585 (0.7%)	63/10,494 (0.6%)	1.16 (0.69-1.96)	0.581	1.08 (0.71–1.66)	0.719
Past and current smoker (n=2,666)						
< 90 days	550/564 (97.5%)	2,062/2,102 (98.1%)	1		1	
90 - 365 days	8/564 (1.4%)	19/2,102 (0.9%)	1.58 (0.69-3.63)	0.281	1.43 (0.68–2.98)	0.345
> 365 days	6/564 (1.1%)	21/2,102 (1.0%)	1.07 (0.43-2.67)	0.883	0.92 (0.44–1.92)	0.823
Alcohol consumption <1 time a week (n=12,420)						
< 90 days	2,495/2,537 (98.3%)	9,729/9,883 (98.4%)	1		1	
90 - 365 days	26/2,537 (1.0%)	95/9,883 (1.0%)	1.07 (0.69-1.65)	0.766	1.02 (0.72–1.46)	0.906
> 365 days	16/2,537 (0.6%)	59/9,883 (0.6%)	1.06 (0.61-1.84)	0.841	0.98 (0.63–1.52)	0.917
Alcohol consumption ≥1 time a week (n=3,325)						
< 90 days	597/612 (97.5%)	2,650/2,713 (97.7%)	1		1	
90 - 365 days	7/612 (1.1%)	38/2,713 (1.4%)	0.82 (0.36-1.84)	0.627	0.68 (0.37–1.28)	0.232
> 365 days	8/612 (1.3%)	25/2,713 (0.9%)	1.42 (0.64-3.17)	0.390	1.16 (0.61–2.22)	0.648
SBP < 140 mmHg and DBP < 90 mmHg (n=11,670)						
< 90 days	2,360/2,403 (98.2%)	9,113/9,267 (98.3%)	1		1	
90 - 365 days	25/2,403 (1.0%)	96/9,267 (1.0%)	1.01 (0.65-1.57)	0.980	0.90 (0.63–1.29)	0.564
> 365 days	18/2,403 (0.7%)	58/9,267 (0.6%)	1.20 (0.70-2.04)	0.504	1.06 (0.69–1.63)	0.801
SBP ≥ 140 mmHg or DBP ≥ 90 mmHg (n=4,075)						
< 90 days	732/746 (98.1%)	3,266/3,329 (98.1%)	1		1	
90 - 365 days	8/746 (1.1%)	37/3,329 (1.1%)	0.97 (0.45-2.08)	0.928	1.04 (0.57–1.90)	0.898
> 365 days	6/746 (0.8%)	26/3,329 (0.8%)	1.03 (0.42-2.51)	0.948	1.05 (0.52–2.10)	0.899
Fasting blood glucose < 100 mg/dL (n=10,584)						
< 90 days	2,167/2,200 (98.5%)	8,281/8,384 (98.8%)	1		1	
90 - 365 days	19/2,200 (0.9%)	67/8,384 (0.8%)	1.08 (0.65-1.81)	0.758	1.01 (0.66–1.55)	0.953
> 365 days	14/2,200 (0.6%)	36/8,384 (0.4%)	1.49 (0.80-2.76)	0.209	1.31 (0.78–2.20)	0.313
Fasting blood glucose ≥ 100 mg/dL (n=5,161)						
< 90 days	925/949 (97.5%)	4,098/4,212 (97.3%)	1		1	
90 - 365 days	14/949 (1.5%)	66/4,212 (1.6%)	0.94 (0.53-1.68)	0.834	0.88 (0.56–1.38)	0.572
> 365 days	10/949 (1.1%)	48/4,212 (1.1%)	0.92 (0.47-1.83)	0.819	0.88 (0.52–1.48)	0.636
Total cholesterol < 200 mg/dL (n=7,977)						
< 90 days	1,618/1,640 (98.7%)	6,225/6,337 (98.2%)	1		1	
90 - 365 days	11/1,640 (0.7%)	62/6,337 (1.0%)	0.68 (0.36-1.30)	0.246	0.61 (0.37–1.00)	0.049*
> 365 days	11/1,640 (0.7%)	50/6,337 (0.8%)	0.85 (0.44-1.63)	0.618	0.74 (0.44–1.24)	0.258
Total cholesterol ≥ 200 mg/dL (n=7,768)						
< 90 days	1,474/1,509 (97.7%)	6,154/6,259 (98.3%)	1		1	
90 - 365 days	22/1,509 (1.5%)	71/6,259 (1.1%)	1.29 (0.80-2.09)	0.295	1.27 (0.85–1.90)	0.244
> 365 days	13/1,509 (0.9%)	34/6,259 (0.5%)	1.60 (0.84-3.03)	0.152	1.62 (0.94–2.80)	0.084

Hemoglobin ≥ 12 g/dL for men and > 10 g/dL for women (n=301)						
< 90 days	71/75 (94.7%)	210/226 (92.9%)	1		1	
90 - 365 days	3/75 (4.0%)	8/226 (3.5%)	0.75 (0.08-6.85)	0.802	0.82 (0.10–6.45)	0.848
> 365 days	1/75 (1.3%)	8/226 (3.5%)	1.51 (0.13-16.9)	0.739	1.76 (0.18–16.8)	0.625
Hemoglobin < 12 g/dL for men and < 10 g/dL for women (n=15,444)						
< 90 days	2,793/3,074 (90.9%)	11,181/12,370 (90.4%)	1		1	
90 - 365 days	159/3,074 (5.2%)	578/12,370 (4.7%)	1.00 (0.68-1.47)	0.996	0.94 (0.69–1.29)	0.713
> 365 days	122/3,074 (4.0%)	611/12,370 (4.9%)	1.13 (0.71-1.80)	0.607	1.05 (0.72–1.53)	0.801
CCI scores = 0 (n=11,002)						
< 90 days	1,893/2,058 (92.0%)	8,185/8,944 (91.5%)	1		1	
90 - 365 days	98/2,058 (4.8%)	376/8,944 (4.2%)	0.56 (0.30-1.04)	0.067	0.54 (0.34–0.85)	0.008*
> 365 days	67/2,058 (3.3%)	383/8,944 (4.3%)	1.74 (0.97-3.11)	0.063	1.62 (0.99–2.66)	0.054
CCI scores = 1 (n=2,213)						
< 90 days	512/573 (89.4%)	1,442/1,640 (87.9%)	1		1	
90 - 365 days	33/573 (5.8%)	93/1,640 (5.7%)	2.89 (1.25-6.70)	0.013*	3.13 (1.31–7.44)	0.010*
> 365 days	28/573 (4.9%)	105/1,640 (6.4%)	0.83 (0.27-2.52)	0.739	0.85 (0.33–2.20)	0.742
CCI scores ≥ 2 (n=2,530)						
< 90 days	459/518 (88.6%)	1,764/2,012 (87.7%)	1		1	
90 - 365 days	31/518 (6.0%)	117/2,012 (5.8%)	1.18 (0.60-2.34)	0.631	1.00 (0.56–1.79)	0.995
> 365 days	28/518 (5.4%)	131/2,012 (6.5%)	0.52 (0.18-1.47)	0.216	0.45 (0.19–1.03)	0.058
Non dyslipidemia history (n=8,594)						
< 90 days	1,571/1,596 (98.4%)	6,898/6,998 (98.6%)	1		1	
90 - 365 days	13/1,596 (0.8%)	51/6,998 (0.7%)	0.88 (0.25-3.04)	0.837	0.84 (0.32–2.20)	0.730
> 365 days	12/1,596 (0.8%)	49/6,998 (0.7%)	2.93 (0.83-10.4)	0.096	2.57 (0.83–7.97)	0.101
Dyslipidemia history (n=7,151)						
< 90 days	1,293/1,553 (83.3%)	4,493/5,598 (80.3%)	1		1	
90 - 365 days	149/1,553 (9.6%)	535/5,598 (9.6%)	0.91 (0.61-1.37)	0.662	0.99 (0.70–1.38)	0.931
> 365 days	111/1,553 (7.1%)	570/5,598 (10.2%)	0.92 (0.56-1.51)	0.746	0.95 (0.64–1.43)	0.819

Abbreviations: RA, Rheumatoid arthritis; CCI, Charlson Comorbidity Index; SBP, Systolic blood pressure; DBP, Diastolic blood pressure;

* Significance at P < 0.05

† Adjusted for age, sex, income, region of residence, SBP, DBP, fasting blood glucose, total cholesterol, hemoglobin, obesity, smoking, alcohol consumption, dyslipidemia history, and CCI scores.

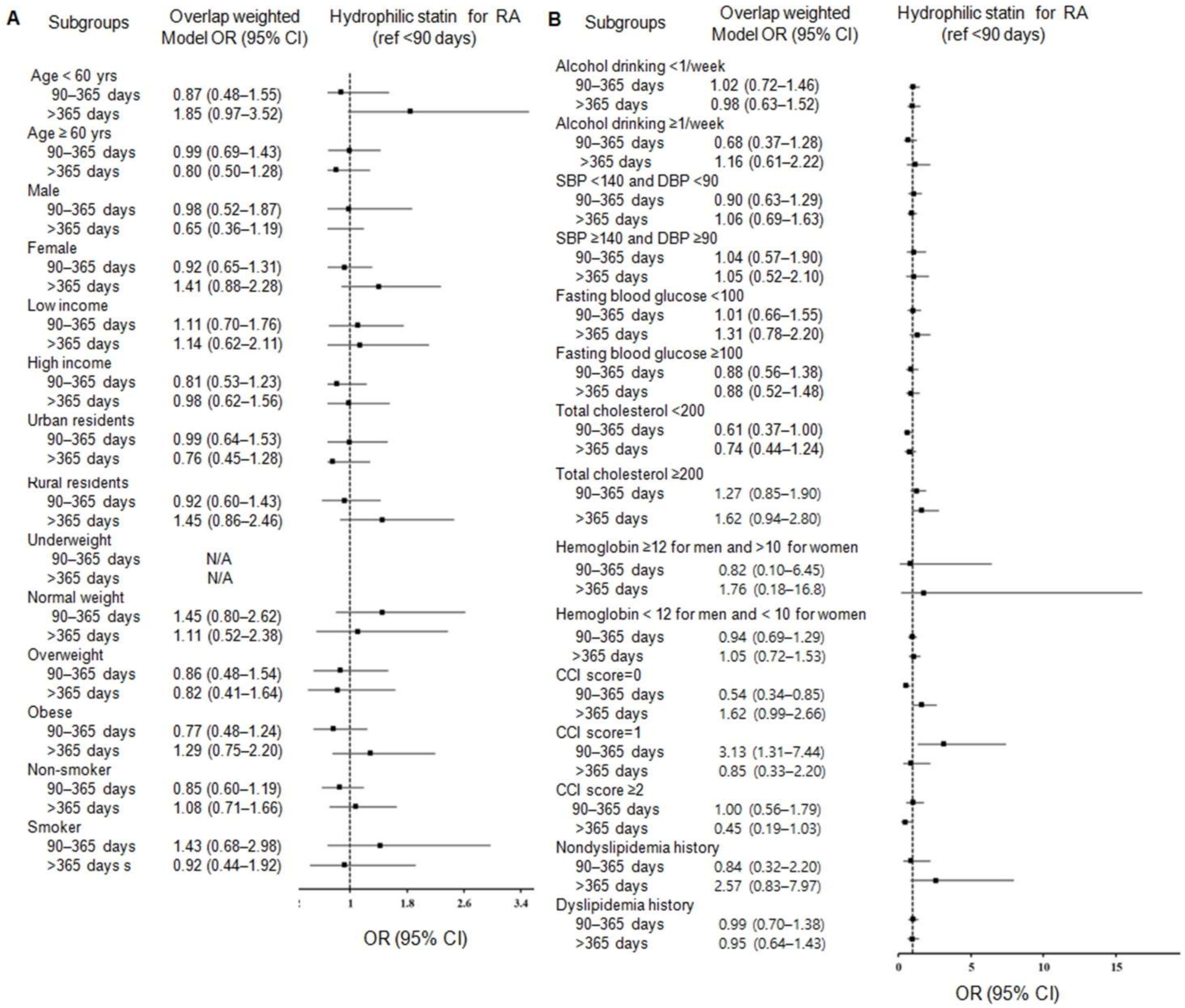

Supplementary Figure S1. Forest plots depicting the association between use duration of hydrophilic statin and a subsequent risk of incident rheumatoid arthritis (RA) in each subgroup.
